# Amino Acid Metabolism and Disease

**DOI:** 10.3390/ijms241511935

**Published:** 2023-07-26

**Authors:** María Ángeles Pajares

**Affiliations:** Department of Structural and Chemical Biology, Centro de Investigaciones Biológicas Margarita Salas (CSIC), Ramiro de Maeztu 9, 28040 Madrid, Spain; mapajares@cib.csic.es

The origin of life is still a matter of debate, and several hypotheses have been proposed to explain how the building blocks leading to the minimal cell were formed [1], with the amino acids among them. However, when considering their role, the first thing that comes to our mind is the fact that amino acids are units that stick together by means of peptide bonds to synthesize proteins [2]. Nevertheless, this is not the single function of amino acids, as they may also serve, among other functions, as substrates for energy production [3,4], as neurotransmitters (Glu and Gly) [5,6], or for the synthesis of a number of compounds that are essential for cell function, such as the methyl donor S-adenosylmethionine and the low-Mr antioxidant glutathione [7]. In order to accomplish this diversity of functions, each amino acid is involved in a network of interconnected pathways, which also allows for the recycling of essential amino acids or provides additional means to synthesize non-essential amino acids in order to guarantee their supply under unfavorable conditions. These conditions may include starvation or malnutrition, as well as diseases of diverse origins (e.g., inborn errors of metabolism or obesity) or aging.

When asked about amino acids, we may also forget those that are not incorporated during protein synthesis, known as non-proteinogenic amino acids [8]. These non-proteinogenic amino acids may be the result of a variety of chemical reactions taking place in the protein sequence during or after folding (posttranslational modifications), whereas others are generated during amino acid metabolism (e.g., homocysteine) and may also have the potential to be used as substrates for protein posttranslational modifications (e.g., S- or N-homocysteinylation) [9,10]. In this line, amino acid-based compounds such as S-adenosylmethionine and glutathione can also serve to modify proteins through enzymatic (e.g., methylations [11]) or non-enzymatic reactions (e.g., S-glutathionylation [12]), respectively. Altogether, these modifications provide the means for the regulation of protein function, degradation, interactions and/or localization, a control that can be exerted through a single modification or through their crosstalk.

The interconnecting pathways of amino acid metabolism may present peculiarities in each organism and/or tissue examined. Specific characteristics of each route are found all throughout the phylogenetic tree and may extend from differences in the oligomeric state of some enzymes [13], their dependence or independence on cofactors [7,14], the restricted expression of certain genes or the reversibility/irreversibility of some of these pathways [7,15], among other causes. Therefore, there are many questions regarding amino acid metabolism that may be specific to a certain cell, system or organism; hence, certain regulatory mechanisms may not be universally extrapolated. The articles included in this Special Issue address some of these aspects using structural approaches, recombinant mutant proteoforms or metabolomics, especially in the context of sulfur amino acid metabolism. This is a part of intermediary metabolism that comprises the methionine cycle and the transsulfuration pathway, which are linked to the folate cycle and the synthesis of glutathione, and in which the key metabolites S-adenosylmethionine, homocysteine and glutathione are synthesized [7].

S-adenosylmethionine is synthesized by the highly conserved family of methionine adenosyltransferases in the first reaction of the methionine cycle [16,17]. This metabolite is best known as the main methyl donor for transmethylation reactions (e.g., epigenetic methylations, synthesis of phosphatidycholine or neurotransmitters) [11], despite its ability to donate many other groups in reactions that are mainly carried out in bacteria [18]. As a result of transmethylation, S-adenosylhomocysteine is produced and hydrolyzed into adenosine and homocysteine, the latter representing a branch point in sulfur amino acid metabolism. As such, homocysteine can be diverted to methionine recycling, trans-sulfuration or exported to the plasma, where high levels of this amino acid are associated with cardiovascular disease [19], thus casting it into the spotlight. Additionally, homocysteine can be used to posttranslationally modify proteins, impairing their function [9]. Transsulfuration allows for the synthesis of cysteine from homocysteine in two reactions, the first of which is catalyzed by cystathionine β-synthase and establishes the link with glutathione synthesis. This special tripeptide occurs at millimolar concentrations within cells and is key to maintaining the redox potential of different subcellular compartments. Additionally, in mammals, the ratio between its reduced and oxidized forms (GSH/GSSG) regulates S-adenosylmethionine synthesis, whose levels also regulate transsulfuration [7].

Examining sulfur amino acid metabolism, we can find a collection of examples of the peculiarities mentioned above, some of which are analyzed in this Special Issue. The articles by Conter et al. [20] and Sánchez-Pérez and Pajares [21] explore the analysis of structural features and their impact on the function of cystathionine β-synthase and methionine adenosyltransferase MAT I enzymes of diverse origin, respectively. Structural knowledge of enzymes is required to gather deep insights into aspects such as the organization of domains and the potential modification of their orientation during catalysis or through the binding of regulators. Moreover, isoenzymes of different organisms may show subtle or greater variations in their domains and/or their regulatory functions. The paper by Conter et al. examines these potential differences between human and *T. gondii* cystathionine β-synthases [20]. These B_6_-dependent enzymes of the transsulfuration pathway are oligomers that catalyze the conversion of homocysteine plus serine into cystathionine and are allosterically regulated by S-adenosylmethionine. Cystathionine β-synthase is also able to synthesize the gasotransmitter H_2_S when using cysteine instead of serine. All cystathionine β-synthases are organized in a catalytic core and a C-terminal module, where S-adenosylmethionine is bound. This module precludes the entrance of substrates into the catalytic site of the human enzyme, but binding of S-adenosylmethionine or removal of this module render the activated form of cystathionine β-synthase. The published crystal structures of human and *T. gondii* cystathionine β-synthases have shown similar assemblies for both isoenzymes, leading to the proposal of a similar regulatory mechanism for the C-terminal module. In contrast, here, Conter et al. report on the lack of consequences of the elimination of the C-terminal module using several procedures, their data suggesting that the regulation of *T. gondii* cystathionine β-synthase differs from that shown by the human enzyme and may putatively involve other adenosyl derivatives for its activation.

Many proteins need to associate to exert their functions, as their active sites may be located between subunits. This is the case for several enzymes involved in amino acid metabolism. Of particular interest is the case of methionine adenosyltransferases, the enzymes that synthesize S-adenosylmethionine. These enzymes use methionine and ATP in a two-step reaction that renders first S-adenosylmethionine and triphosphate, the latter being further hydrolyzed into pyrophosphate and inorganic phosphate. The active site of methionine adenosyltransferases is located at the interface between two catalytic subunits that are associated in such a way that the homodimer has two active sites opposite one to another [13]. Hence, the minimum active form of the enzyme is the homodimer. Interestingly, the main gene expressed in the adult mammalian liver is *MAT1A*, which codifies for the MATα1 catalytic subunit, which assembles both as homodimers (MAT III) and homotetramers (MAT I) that differ in their kinetic properties. MAT I has a much higher affinity for methionine than MAT III, and hence, a decrease in MAT I levels in liver diseases has a negative impact on the production of S-adenosylmethionine. Therefore, understanding the mechanisms through which the MAT I/MAT III ratio is controlled remains a subject of interest in this field. The article by Sánchez-Pérez and Pajares analyzes the polar interactions identified between homodimers in the rat MAT I structure to offer insights into their importance in the stability and kinetic properties of the homotetramer [21]. Using structural data and site-directed mutagenesis, the authors demonstrated that a few polar interactions involving three residues of the central domain in MATα1 monomers are enough to maintain the MAT I homotetramer assembly.

Continuing with sulfur amino acid metabolism, the paper by Borowczyk and Glowacki addresses the impact of a common aesthetic procedure [22]. As we all know, we humans are very much worried about our appearance, and hence, we subject our bodies to a number of cosmetic procedures that may affect our tissues. Borowczyk and Glowacki analyzed the impact that the use of hybrid UV manicure for six months has in nail plates, specifically in regard to their contents of the sulfur amino acids cysteine and methionine [22]. This common procedure combines the use of acetone or a mechanical treatment to remove previous layers of the polish with the application of a varnish that is cured using UV radiation. These authors established an optimized protocol to evaluate cysteine and methionine contents in nail plates in order to further compare their levels in samples obtained before and after the cosmetic treatment. Their results indicated a significant decrease in the levels of both amino acids, with that for methionine being greater than that for cysteine, as well as a reduced thickness of nail plates subjected to hybrid manicures. Since the nail plates are responsible for nourishing the nails, these data suggest that this procedure may affect the production and/or degradation of cysteine-rich proteins, such as keratins, that are main components of nails, in turn altering their physical properties and their function.

The last group of articles in this Special Issue have a wider objective regarding health conditions. Mir et al. analyzed changes in amino acid levels in subjects with obesity [23], while Lv et al. reviewed these alterations in aging and associated osteoporosis [24]. Obesity is a condition with increasing incidence in the population that is associated with multiple pathologies. It may present with or without metabolic disorders, and the two types of obesity differ in their lipid profiles and insulin sensitivity and, in turn, in their association with other pathologies. Therefore, there is great interest in the analysis of the metabolomic profiles of subjects with either type of obesity to decipher the metabolic routes that are affected in each case, as well as the putative differences in the alterations of their regulatory mechanisms. The work presented in this Special Issue by Mir et al. evaluated the serum metabolomic profiles of age-matched subjects with obesity or obesity with metabolic syndrome [23]. This untargeted-metabolomic study analyzed nearly 700 metabolites in these samples and found differences between the two groups in approximately 80 of them. Of note, decreases in 15 amino acids were detected in the sera of the obesity with metabolic syndrome cohort compared to the obesity subjects, including, e.g., tryptophan and its metabolite kynurenine. Pathway enrichment indicated that altered pathways between the two types of obesity subjects include lysine degradation, arginine and proline metabolism.

A normal condition that every organism undergoes until dead is aging, which is associated with several health problems, including e.g., osteoporosis. The review by Lv et al. summarizes the knowledge on the role of essential amino acids in bone metabolism obtained to date, both in vitro and in vivo [24]. Moreover, the authors discuss the fluctuations in these types of amino acids described in the sera of subjects with osteoporosis, as well as data on the impact of supplements with essential amino acids on several characteristics of the bone. Additionally, it is shown that amino acids such as lysine, threonine, methionine, tryptophan and isoleucine improve osteoclast behavior, increasing proliferation and differentiation. The authors also discuss the effects of different oxidation products of tryptophan on bone marrow stem cells, favoring their differentiation into either osteoclasts or adipocytes. The review concludes that essential amino acids are important during aging because of their nutritional and regulatory roles and that supplements containing essential amino acids seem, in general, to improve bone mass density, despite the small size of the cohorts analyzed.

Finally, I hope that the readers will enjoy the articles collected in this Special Issue, which due to their range of topics, can only offer a flavor of the many unknowns remaining in the field of amino acid metabolism and the diversity of enzyme behaviors and/or regulatory mechanisms. I also hope that the following Special Issue on “Amino Acid Metabolism and Disease 2.0” will continue to enhance our understanding of the roles and impact of amino acids on human physiology and pathophysiology, as well as to analyze the diversity observed among organisms in the pathways and enzymes involved.
